# Effect of protease supplementation on apparent ileal crude protein and amino acid digestibility of over-processed soybean meals in broilers

**DOI:** 10.1186/s40104-022-00728-w

**Published:** 2022-07-11

**Authors:** Sergio Salazar-Villanea, Maikol Astúa-Ureña, Allison Masís-Montoya, Juan I. Herrera-Muñoz, Catalina Salas-Durán

**Affiliations:** 1 Escuela de Zootecnia, Universidad de Costa Rica, San José, Costa Rica; 2 Centro de Investigación en Nutrición Animal, Universidad de Costa Rica, San José, Costa Rica

**Keywords:** Amino acids, Apparent ileal digestibility, Broilers, Exogenous protease

## Abstract

**Background:**

Nutritional value of proteins in feed ingredients can be negatively affected by hydrothermal processing, which causes large variation in the bioavailability of amino acids (AA) and negatively affects animal productive performance. Supplementation of exogenous proteases could increase the rate of digestion of damaged proteins, thereby increasing overall AA digestibility and bioavailability. The aim was to determine the effect of exogenous protease supplementation on the apparent ileal digestibility (AID) of crude protein (CP) and AA of soybean meals (SBM) with different degrees of hydrothermal processing in broilers.

**Methods:**

The experiment involved a 3 × 2 factorial arrangement, with SBM processing time (commercial SBM or autoclaved for 30 or 60 min at 120 °C) and protease supplementation (not supplemented and supplemented) as factors. Protease was included at three times the recommended dose (0.06%) and the experimental diets were fed from 15 to 21 d.

**Results:**

The interaction between the effects of SBM processing and protease supplementation was significant for the AID of CP (*P* = 0.01), Trp (*P* = 0.01), Gly (*P* = 0.03) and Pro (*P* = 0.03), and also for the average daily gain (*P* = 0.01) and feed conversion ratio (*P* = 0.04). Increasing the processing time of SBM decreased (*P* < 0.0001) the AID of all amino acids, whilst the effect of protease supplementation was only significant for the AID of Phe (*P* = 0.02) and Tyr (*P* = 0.01).

**Conclusions:**

Exogenous protease supplementation at three times the commercial dose does not seem to offset the negative effects of hydrothermal processing of SBM on the apparent ileal digestibility of CP and amino acids or performance of broilers. Whilst positive numerical improvements of digestibility and performance (ADG and FCR) were noticed with protease supplementation at relatively mild processing levels, negative results were obtained with the harsh-processed meals.

## Background

The nutritional value of feed ingredients can be negatively affected by hydrothermal processing. Protein denaturation during processing could lead to changes of their structural conformation, resulting in the formation of protein aggregates, which render the hydrolytic sites inaccessible to enzymes [1, 2]. In addition, processing can induce chemical modifications to the structure of amino acids (AA) resulting in the formation of Maillard reaction products [3–5]. The chemically modified AA cause a stearic hindrance effect, which also limits enzyme accessibility for hydrolysis [6]. The main protein-bound AA affected, lysine and arginine, are also the target AA of trypsin, one of the main proteolytic endogenous enzymes, which likely reduces the efficiency of hydrolysis [2]. The overall result is a reduction in the rate of protein hydrolysis [7, 8], which results in a decrease of protein digestibility [8]. Fermentation of undigested proteins can result in undesired putrefaction products in the gut (e.g. biogenic amines, phenolic and indolic compounds) and increase the proliferation of pathogenic bacteria [9]. Moreover, undigested proteins contribute to N emissions to the environment and can therefore be considered as a pollutant [10].

Exogenous enzymes, such as proteases, are a tool in state-of-the-art animal nutrition to increase the nutritional value of feed ingredients and improve animal performance at a lower cost [11]. The proteolytic mechanism of exogenous proteases is complementary to that of endogenous proteases [12]. For example, trypsin, one of the main endogenous digestive enzymes, is highly specific for Lys and Arg; whilst, the exogenous subtilisin-like proteases have high affinities towards large hydrophobic amino acids, such as Phe and Tyr [13]. Therefore, an increase in the rate of hydrolysis and amino acid digestibility could be expected when exogenous proteases are supplemented in the diets [14].

Our objective was to determine the effect of exogenous protease supplementation on the productive performance and apparent ileal digestibility (AID) of crude protein (CP) and AA of soybean meals (SBM) with different degrees of hydrothermal processing. We hypothesized that exogenous protease supplementation would increase the rate of hydrolysis of thermally damaged proteins, thereby increasing AA digestibility at the end of the small intestine.

## Methods

### Experimental setup

Animal experimental procedures were approved by the Animal Care and Use Committee, University of Costa Rica, under the authorization number CICUA-110-17. The experiment involved a 3 × 2 factorial arrangement, with SBM autoclaving time (commercial SBM or autoclaved for 30 or 60 min at 120 °C) and protease supplementation (not supplemented and supplemented; RONOZYME ProAct, Novozymes, Krogshoejvej, Denmark) as factors. This design resulted in 6 experimental diets: unautoclaved SBM without and with enzyme supplementation (SBM and SBM + E, respectively), 30 min autoclaved SBM without and with enzyme supplementation (SBM30 and SBM30 + E, respectively) and 60 min autoclaved SBM without and with enzyme supplementation (SBM60 and SBM60 + E, respectively).

### Experimental diets

Commercial SBM was purchased (Concentrados Gastón Fernández, Cartago, Costa Rica) and divided in 3 batches: one was left unautoclaved, whilst the other two were autoclaved at 120 °C for 30 and 60 min, respectively. Temperatures during autoclaving were measured with internal thermocouples and ranged from 118 to 123 °C.

Experimental diets were formulated according to the recommendations from Ravindran et al. [15] for the evaluation of amino acid digestibility of raw materials in broilers (Table 1) and fed as mash. Chromium oxide (Cr_2_O_3_) was included in the experimental diets as a marker. The exogenous protease was supplemented at 3 times the commercial recommendation (commercial recommendation is 15,000 PROT/kg), in order to assure that the enzyme was not a limiting factor for hydrolysis. One PROT unit is defined as the amount of serine protease that liberates 1 μmol para-nitroaniline (pNA) from 1 mmol/L Suc-Ala-Ala-Pro-PhepNA (C_30_H_36_N_6_O_9_) (Sigma-Aldrich, St. Louis, MO, USA) substrate per minute at pH = 9.0 and at 37 °C. The analyzed nutritional composition of the experimental diets is depicted in Table 2.

**Table 1:** Ingredient and nutritional composition of the experimental diets, g/kg as is

Ingredients	Basal diet	Basal diet + enzyme
Dextrose^a^	507	506.4
Soybean meal^b^	416	416
Soybean oil	40	40
Dicalcium phosphate	19	19
Calcium carbonate	10	10
Cr_2_O_3_	3	3
Sodium bicarbonate	2	2
Salt	2	2
Vitamins and minerals premix^c^	1	1
Enzyme^d^	–	0.6
Nutritional composition (calculated)		
Metabolizable energy, kcal/kg	3110	3107
Crude protein	196.10	196.10
Available phosphorus	5.02	5.02
Calcium	8.49	8.49

^a^Fufeng Group, China

^b^Commercial soybean meal used was unautoclaved, autoclaved 30 min at 120 °C or autoclaved 60 min at 120 °C

^c^Vitamins and minerals supplied per kg of diet: Cu (sulfate), 12 mg; Fe (sulfate), 40 mg; I (iodide), 1 mg; Se (selenate), 0.30 mg; Mn (sulfate and oxide), 80 mg; Zn (sulfate and oxide), 80 mg; retinol, 10,000 UI; cholecalciferol, 3000 UI; tocopheryl acetate, 35 UI; menadione, 2.50 mg; thiamine, 1.75 mg; riboflavin, 6.50 mg; niacin, 55 mg; pantothenate 10.70 mg; pyridoxine, 3.60 mg; folate, 1.50 mg; cyanocobalamin, 15 μg; biotin, 110 μg

^d^RONOZYME ProAct, Novozymes, Krogshoejvej, Denmark

**Table 2:** Analyzed nutrient composition of the soybean meals and experimental diets, g/kg as is

Nutrient	SBM^a^ – autoclaving time			Experimental diets					
	0 min	30 min	60 min	SBM	SBM + E	SBM30	SBM30 + E	SBM60	SBM60 + E
DM	868.0	856.4	847.5	880.9	877.5	882.5	875.8	874.7	869.9
CP	441.6	437.8	437.9	192.3	192.5	192.9	205.4	209.8	215.8
Cr	–	–	–	0.040	0.030	0.070	0.043	0.075	0.079
Protease (PROT/kg)	–	–	–	nd	56,470	LOQ	48,680	nd	53,900
Essential amino acids									
Arg	27.9	26.4	24.8	12.9	10.9	11.6	16.5	10.3	12.7
His	13.7	10.2	12.7	6.0	5.3	5.9	8.0	5.6	6.5
Ile	18.0	17.6	17.7	8.6	7.6	8.5	11.2	8.2	9.3
Leu	30.4	29.3	28.8	13.7	12.5	13.6	18.5	13.4	15.1
Lys	24.8	22.4	20.6	10.1	8.4	8.8	12.5	7.9	8.8
Met	6.1	6.2	5.9	2.9	2.8	3.1	3.5	2.8	2.9
Phe	23.6	19.7	22.1	10.5	9.6	9.5	14.4	9.9	11.7
Thr	15.3	15.1	15.0	7.2	6.5	7.2	9.6	7.1	8.0
Trp	7.6	6.7	6.5	2.9	2.2	3.9	2.2	2.5	1.9
Val	18.4	17.8	17.8	8.6	7.8	8.3	11.2	8.2	9.2
Non-essential amino acids									
Ala	16.2	15.3	15.6	7.4	6.8	7.6	10.0	7.3	7.9
Asx	45.8	44.1	44.6	22.3	19.5	21.9	29.1	21.1	24.1
Cys	6.1	5.5	4.9	2.8	2.6	3.1	3.3	2.5	2.5
Glx	71.2	70.8	66.5	32.7	29.5	32.2	43.6	31.8	35.0
Gly	14.2	14.2	14.1	6.8	6.1	6.7	9.0	7.1	7.4
Pro	20.9	18.9	20.0	9.0	8.7	8.5	12.6	8.8	9.8
Ser	18.4	18.5	17.5	8.1	7.6	8.2	11.2	8.3	9.3
Tyr	12.0	13.0	12.5	5.2	4.8	5.4	7.8	4.5	5.9

^a^*Abbreviations: SBM* soybean meal, *E* enzyme, *DM* dry matter, *CP* crude protein, *Cr* chromium content, *nd* not detected, *LOQ* below limit of quantification

### Animals and housing

A total of 504 one-day-old Cobb 500 broilers were allocated at a density of 12 birds/m^2^ in an open environment facility. Temperatures during the experiment averaged 22.84 ± 2.61 °C and a light regimen of 12 h light and 12 h dark was used. There were 7 repetitions per treatment for the experimental diets, for a total of 42 experimental units, which were allotted to a total of 6 experimental blocks, where each experimental diet was represented at least once in every experimental block. Each pen was considered as an experimental unit. Birds had ad libitum access to water and a commercial pre-starter (1–7 d) and starter diets (8–14 d). From d 15–21, the birds received ad libitum access to the experimental diets. Average daily gain (ADG) of each bird was calculated using the average weight at the start and the end of the experimental period (14 and 21 d, respectively), divided by the number of birds in each experimental unit. Average daily feed intake (ADFI) was determined as the difference between the feed offered during the 14–21 d period and feed refusal at 21 d and calculated per bird. Feed conversion ratio (FCR), corrected for mortality, was calculated as the ratio between ADFI and ADG.

### Sample collection and chemical analysis

On d 21, 6 birds were randomly selected per pen and euthanized by cervical dislocation. Digestive content from the last 20 cm of the ileum (anterior to the ileo-cecal valve) of each bird was collected by gentle stripping and pooled per experimental unit. Samples were immediately frozen and kept at − 70 °C, followed by freeze-drying and grinding to pass a 1-mm sieve. Chromium content was determined by inductively coupled plasma-mass spectrometry (iCAP™ RQ ICP-MS, Thermo Fisher Scientific, Waltham, MA) following microwave digestion of the samples with nitric acid [16, 17]. Nitrogen content of the diets and the freeze-dried digesta was determined by combustion using a nitrogen analyzer (Rapid N Exceed, Elementar, Langenselbold, Germany) and the CP content was calculated using a 6.25 conversion factor. Amino acid contents were determined by ion exchange chromatography using post-column derivatization with ninhydrin in an amino acid analyzer (Hitachi L-8900, Tokyo, Japan), after in vacuo hydrolysis with 6 mol/L HCl and 1% phenol at 110 °C for 24 h [18, 19], using norleucine as an internal standard. For the analysis of Met and Cys, the samples were oxidized overnight at 2 °C using performic acid before hydrolysis [20]. Tryptophan content was determined after in vacuo alkaline hydrolysis (4.2 mol/L NaOH) at 110 °C for 24 h [21]. Serine protease activity in the experimental diets was determined using a colorimetric method described by Yasar [22], where the amount of yellow complex released by serine protease enzyme from the substrate “Suc-Ala-Ala-Pro-Phe-pNA (C_30_H_36_N_6_O_9_)” at pH = 9.0 and at 37 °C is related to the enzymatic activity measured at 405 nm, using a standard curve of a certified Ronozyme ProAct™ serine protease standard. The limit of quantification of the method was 1000 PROT/kg.

### Calculations and statistical analysis

Apparent ileal digestibility of CP and AA was calculated according to the following equation (Eq. 1):

1 $$ \mathrm{AID}\ \left(\%\right)=\left[1-\left({X}_d/{X}_f\right)\times \left({Cr}_f/{Cr}_d\right)\right]\times 100 $$

where *X*_*d*_ is the concentration of CP or amino acid in the ileal digesta (g/kg of DM), *X*_*f*_ is the concentration of CP or amino acid in the experimental diet (g/kg of DM), *Cr*_*f*_ is the concentration of chromium in the experimental diet (g/kg of DM) and *Cr*_*d*_ is the concentration of chromium in the ileal digesta (g/kg of DM).

The AID of CP and AA were statistically analyzed using a two-way ANOVA procedure of SAS software, Version 9.4 m6 (SAS Institute Inc., Cary, NC). The model included the effects of the experimental block, SBM processing, protease supplementation and the interaction between SBM processing and protease supplementation. Significance was considered at *P*-values < 0.05 and tendencies were declared at *P*-values between 0.05–0.10. *Post-hoc* comparisons were performed using the Tukey-Kramer adjustment.

## Results

The interaction between the effects of SBM processing and protease supplementation was significant for the AID of CP (*P* = 0.01), Trp (*P* = 0.01), Gly (*P* = 0.03) and Pro (*P* = 0.03). The interaction effect on CP, Gly and Pro responds to a numerical increase in the AID of SBM and SBM30 diets with protease supplementation, though a reduction in the SBM60 diets. In addition, other amino acids (Ile, Leu, Lys, Met, Phe, Val, Ala, Cys, and Glu) exhibited a similar response, which was shown as a tendency for a significant effect of the interaction between SBM processing and protease supplementation. One exception to this trend was the AID of Trp, which for the SBM30 diet already showed a decrease of −6.34%, whilst the decrease for the SBM60 diet reached −27.37%.

Increasing the processing time of SBM decreased (*P* < 0.0001) the AID of all amino acids (Table 3). The decrease in CP digestibility reached −13.4% and −37.3% for the SBM30 and SBM60 diets, respectively, compared to the SBM diets. The average AID of essential amino acids decreased from 88% in the SBM diets to 82% and 60% in the SBM30 and SBM60 diets, respectively. The largest decrease in digestibility for the essential amino acids after the hydrothermal treatment was present for Lys, which was reduced by −11.9% and −46.96% comparing the SBM30 and SBM60 diets with the SBM diet, respectively. The average AID of the non-essential amino acids decreased from 85% in the SBM diets to 75% and 46% in the SBM30 and SBM60 diets, respectively. For the non-essential amino acids, the largest decrease after the hydrothermal treatment was present for Cys, which was reduced by −14.1% and −64.3% comparing the SBM30 and SBM60 diets with the SBM diet, respectively.

**Table 3:** Apparent ileal digestibility of crude protein (CP) and amino acids, %

Nutrient	SBM processing			Protease		Experimental diets							*P-*value		
	SBM	SBM30	SBM60	No	Yes	SBM	SBM + E	SBM30	SBM30 + E	SBM60	SBM60 + E	SEM	P	E	P × E
CP	85.47	72.05	48.19	68.21	68.93	82.36^ab^	88.59^a^	67.23^bc^	76.87^ab^	55.05^cd^	41.33^d^	10.62	< 0.0001	0.83	0.01
Essential amino acids															
Arg	93.25^x^	89.06^x^	75.74^y^	84.67	87.36	91.54	94.95	86.51	91.61	75.96	75.52	5.07	< 0.0001	0.10	0.35
His	90.24^x^	82.61^y^	61.88^z^	76.71	79.78	87.98	92.50	78.53	86.69	63.63	60.14	7.39	< 0.0001	0.19	0.12
Ile	87.55^x^	82.67^x^	64.84^y^	76.67	80.03	84.51	90.59	78.81	86.53	66.70	62.97	7.17	< 0.0001	0.14	0.09
Leu	87.68^x^	84.26^x^	68.95^y^	78.53	82.07	84.47	90.90	80.63	87.89	70.48	67.41	6.59	< 0.0001	0.10	0.09
Lys	89.76^x^	77.86^y^	42.80^z^	68.68	71.59	87.42	92.11	72.19	83.52	46.44	39.15	10.71	< 0.0001	0.39	0.09
Met	90.96^x^	84.29^y^	66.32^z^	80.20	80.85	88.61	93.31	82.12	86.47	69.87	62.78	7.00	< 0.0001	0.77	0.06
Phe	91.53^x^	89.46^x^	80.36^y^	85.53	88.71	89.03	94.03	86.57	92.36	81.00	79.73	4.27	< 0.0001	0.02	0.08
Thr	81.23^x^	71.04^x^	42.22^y^	62.22	67.44	77.16	85.31	64.67	77.41	44.83	39.60	12.05	< 0.0001	0.17	0.14
Trp	82.41	74.25	39.15	69.86	60.68	79.33^a^	85.49^a^	77.42^a^	71.08^ab^	52.83^b^	25.46^c^	13.72	< 0.0001	0.04	0.01
Val	86.84^x^	80.88^x^	61.23^y^	74.56	78.07	83.58	90.10	76.56	85.19	63.55	58.91	7.92	< 0.0001	0.17	0.08
Non-essential amino acids															
Ala	86.28^x^	79.36^x^	55.59^y^	72.13	75.36	82.96	89.60	74.82	83.91	58.60	52.58	9.14	< 0.0001	0.26	0.08
Asp	86.73^x^	69.56^y^	30.80^z^	60.45	64.28	83.92	89.54	62.95	76.16	34.47	27.13	13.09	< 0.0001	0.35	0.13
Cys	77.66^x^	63.55^x^	13.36^y^	50.30	52.75	70.78	84.54	58.79	68.30	21.31	5.40	16.67	< 0.0001	0.64	0.06
Glu	90.81^x^	82.44^y^	60.54^z^	76.99	78.87	88.85	92.78	78.38	86.51	63.74	57.33	7.63	< 0.0001	0.43	0.05
Gly	82.22	69.09	37.73	61.11	64.92	77.96^ab^	86.49^a^	61.92^bc^	76.26^ab^	43.44^cd^	32.01^d^	12.60	< 0.0001	0.34	0.03
Pro	86.72	77.04	50.25	68.80	73.87	82.82^ab^	90.61^a^	70.08^b^	84.00^ab^	53.49^c^	47.00^c^	13.56	< 0.0001	0.10	0.03
Ser	84.26^x^	75.34^x^	51.45^y^	67.80	72.90	80.47	88.06	69.12	81.57	53.82	49.08	10.32	< 0.0001	0.12	0.10
Tyr	89.02^x^	85.46^x^	66.78^y^	77.83	83.01	85.02	93.02	81.98	88.94	66.50	67.06	6.13	< 0.0001	0.01	0.24

*Abbreviations*: *SEM* standard error of the mean, *P* soybean meal processing, *E* enzyme supplementation

^a,b,c,d^Means with different superscript letters within a row are significantly different (*P* < 0.05)

^x,y,z^Means with different superscript letters within a row are significantly different (*P* < 0.05)

The effect of protease supplementation was only significant for the AID of Phe (*P* = 0.02) and Tyr (*P* = 0.01). Protease supplementation increased the AID of Phe and Tyr in +3.2% and +5.2%, respectively, whilst for Trp there was a decrease of −9.18%.

Productive performance of the birds during the 14–21 d period is presented in Table 4. Overall mortality during the experiment was 1%. The effect of protease supplementation was not significant for any of the productive parameters measured (*P* > 0.05). The ADFI of the birds consuming the SBM diets (76.96 g/d) was higher (*P* = 0.02) than that of the birds consuming the SBM60 diets (73.43 g/d), whilst the birds consuming the SBM30 diets had an intermediate ADFI (76.36 g/d). The interaction between SBM processing and protease supplementation was significant for the ADG (*P* = 0.01) and the FCR (*P* = 0.04). Similar to the interaction for the AID of CP and some amino acids, the interaction between SBM processing and protease supplementation for ADG and FCR originates in a numerical improvement after protease supplementation in the SBM30 diets, almost reaching the levels for the SBM diets, whilst a decrease after supplementation to the SBM60 diets. The effects on FCR are mainly originating from differences in ADG.

**Table 4:** Productive performance from 14 to 21 days of the broilers fed with the experimental diets

Parameter	SBM processing			Protease		Experimental diets						SEM	*P*-value		
	SBM	SBM30	SBM60	No	Yes	SBM	SBM + E	SBM30	SBM30 + E	SBM60	SBM60 + E		P	E	P × E
ADFI^1^, g/d	76.96^x^	76.36^xy^	73.43^y^	75.95	75.22	77.01	76.91	75.96	76.76	74.86	71.99	3.34	0.02	0.49	0.34
ADG, g/d	47.78	44.17	38.83	43.67	43.52	48.53^a^	47.01^ab^	42.27^bc^	46.08^ab^	40.21^cd^	37.45^d^	2.79	< 0.0001	0.86	0.01
FCR	1.61	1.73	1.90	1.75	1.75	1.59^d^	1.64^cd^	1.80^abc^	1.66^bcd^	1.86^ab^	1.95^a^	0.12	< 0.0001	0.99	0.04

^1^*Abbreviations*: *ADFI* average daily feed intake, *ADG* average daily gain, *FCR* feed conversion ratio, *SEM* standard error of the mean, *P* soybean meal processing, *E* enzyme supplementation

^a,b,c,d^Means with different superscript letters within a row are significantly different (*P* < 0.05)

^x,y^Means with different superscript letters within a row are significantly different (*P* < 0.05)

## Discussion

There is variability in the nutritional quality of SBM, originating in the wide range of processing conditions used in the production plants [23–25]. Lysine contents from different SBM production plants in United States ranged from 6.35 to 6.43 g/100 g CP [23]. In the current study, lysine content in the SBM was 5.62 g/100 g CP, which can be considered as low and closely resembles the lower values reported by Ibáñez et al. [24] and Ravindran et al. [25] in meals from different countries of origin, which could be related to harsh processing conditions during the production of the meal. Recent studies performed in Australia [26], reported SBM with lysine contents averaging 4.82 g/100 g CP. Similar contents can only be found in the SBM that was autoclaved for 60 min in the present study (4.70 g/100 g CP).

The effects of hydrothermal processing are progressively severe on protein solubility and lysine content [27], which reflect on slower rates of protein hydrolysis [8, 28] and decreased CP and amino acid digestibility [8, 29]. Lysine contents in the present study were linearly reduced by 0.015 g/100 g CP for every minute of hydrothermal treatment of the SBM (*r*^2^ = 0.997). The decrease of Lys content after hydrothermal processing was probably due to the formation of advanced Maillard reaction products, which cannot be reversed by acid-hydrolysis during the amino acid analysis, in contrast to Amadori products, that can be partly reversed to Lys [30]. Lysine digestibility was the most affected essential amino acid by the hydrothermal treatment in the present study. For every 1 g/kg loss in Lys content of the soybean meals, there was a decrease of −18.6% of CP digestibility and −23.5% of Lys digestibility (*r*^2^ = 0.97 and 0.93, respectively). This is not surprising, as Lys is one of the target amino acids of the pancreatic proteolytic enzymes (mainly trypsin). In addition, the deleterious effect of autoclaving the SBM for 60 min was especially notorious for the AID of Cys. Harsh conditions during hydrothermal processing cause an increase in the formation of disulfide bonds, especially in the insoluble protein fraction, formed between Cys residues in proteins [31, 32]. The formation of these covalent bonds likely reduced overall accessibility of enzymes for proteolysis and the digestibility of this particular amino acid.

The AID of Phe and Tyr also decreased with increasing autoclaving time of the SBM (*P* < 0.0001). However, in contrast to the rest of the AA, the AID of Phe and Tyr increased by exogenous protease inclusion in the diets (*P* = 0.02 and *P* = 0.01, respectively). Subtilisin-like proteases, such as the one used in the present study, have a high affinity towards large hydrophobic AA, such as Phe and Tyr [13], which explains that these were the only AA affected by inclusion of the exogenous protease across all diets.

The numerical increase in CP digestibility and amino acid digestibility with protease supplementation in the SBM and SBM30 diets contrasts with the observed decrease in the SBM60 diets. There seems to be a threshold where protease supplementation cannot recover the negative effects of thermal protein damage. Furthermore, contrary to what we expected, inclusion of the exogenous protease in the SBM60 diets decreased the digestibility of CP and most AA. Although most studies [33] have reported an increase in CP and amino acid digestibility with the inclusion of exogenous proteases, there is limited information regarding the effects on thermally damaged ingredients. Moreover, the effects of the inclusion of exogenous proteases seem to be dependent on the dose of the enzyme used and the duration of the period of supplementation, as described by Yuan et al. [34]. These authors reported that, after 42 days of supplementation, the inclusion of 40 mg/kg of an acid protease in combination with non-starch polysaccharide hydrolytic enzymes increased the activity of pancreatic trypsin by 27.41%, whilst the inclusion of the protease at levels of 80 and 160 mg/kg (also in combination with the non-starch polysaccharide hydrolytic enzymes) decreased the activity of pancreatic trypsin by 10.75% and 25.88%, respectively. Endogenous enzymatic activity might have been reduced in the present study, as the exogenous protease was included at three times the recommended dose. A reduction of the secretion of endogenous enzymes due to an excess of exogenous enzymes supplementation is likely to affect the digestibility of the substrate, especially when the substrate has suffered extreme physical and chemical modifications due to hydrothermal over-processing. Additionally, Maillard reactions could increase the formation of organic acids [35, 36], which could have reduced the intestinal pH below the optimum for the activity of the exogenous protease. The combination of these three factors: a reduction in endogenous proteolytic activity, a reduction of the optimum pH for the exogenous protease and the profound structural and chemical modifications of the proteins due to hydrothermal processing, are likely to explain the reduction in CP and amino acid digestibility with the inclusion of exogenous enzymes in the SBM60 diet.

The negative effects of hydrothermal damage on CP and amino acid digestibility were also noticeable on the ADG of the birds. However, the influence of the digestibility on ADG does not appear to be linear. For example, whilst a reduction of approximately 13% in the AID of Lys in the SBM30 compared to the SBM diets caused a reduction of 7.5% in the ADG of the birds, a reduction of approximately 50% in the AID of Lys in the SBM60 compared to the SBM diets only caused a reduction of less than 20% of the ADG of the birds. Although it was not determined in the present study, it is possible that catabolism of unbalanced amino acids in the diets with higher degrees of processing resulted in a change of the body composition of the birds, which increased the deposition of body fat. Changes in body composition due to catabolism of unbalanced amino acids have been reported in studies with broilers on the effects of different dietary digestible Lys levels in the diets [37] and studies that tested diets with low-protein contents [38]. It is worth mentioning that the diets in the present study were not formulated to maximize the performance of the birds, but to test the digestibility of SBM as an ingredient. Therefore, the AA content in the diets were likely limiting for growth. Furthermore, the significant interaction between the effects of SBM processing and protease supplementation on ADG and FCR, which shows a positive effect of protease supplementation on the SBM30 diets and a negative effect on the SBM60 diets, is likely to be caused by the AID of CP and most amino acids, which also exhibit a similar pattern for the interaction. Amino acid digestibility directly influences the productive performance of broilers [39].

## Conclusions

In conclusion, exogenous protease supplementation at three times the commercial dose does not seem to offset the negative effects of hydrothermal processing of SBM on the AID of CP and amino acids or performance of broilers. Whilst positive numerical improvements of digestibility and performance (ADG and FCR) were noticed with protease supplementation at relatively mild processing levels, negative results were obtained with the harsh-processed meals.

## Data Availability

All data generated or analyzed during this study are available from the corresponding author upon reasonable request.

## Abbreviations

AA: Amino acids
ADFI: Average daily feed intake
ADG: Average daily gain
AID: Apparent ileal digestibility
CP: Crude protein
FCR: Feed conversion ratio
SBM: Soybean meal
